# Uptake and intracellular accumulation of diamond nanoparticles – a metabolic and cytotoxic study

**DOI:** 10.3762/bjnano.8.165

**Published:** 2017-08-10

**Authors:** Antonín Brož, Lucie Bačáková, Pavla Štenclová, Alexander Kromka, Štěpán Potocký

**Affiliations:** 1 Institute of Physiology of the Czech Academy of Sciences, v.v.i., Vídeňská 1083, 142 20, Praha 4, Czech Republic; 2 Institute of Physics of the Czech Academy of Sciences, v.v.i., Cukrovarnická 10, 162 00 Praha 6, Czech Republic

**Keywords:** cell viability, FTIR, live-cell imaging, MTS, nanodiamond, SAOS-2 cells

## Abstract

Diamond nanoparticles, known as nanodiamonds (NDs), possess several medically significant properties. Having a tailorable and easily accessible surface gives them great potential for use in sensing and imaging applications and as a component of cell growth scaffolds. In this work we investigate in vitro interactions of human osteoblast-like SAOS-2 cells with four different groups of NDs, namely high-pressure high-temperature (HPHT) NDs (diameter 18–210 nm, oxygen-terminated), photoluminescent HPHT NDs (diameter 40 nm, oxygen-terminated), detonation NDs (diameter 5 nm, H-terminated), and the same detonation NDs further oxidized by annealing at 450 °C. The influence of the NDs on cell viability and cell count was measured by the mitochondrial metabolic activity test and by counting cells with stained nuclei. The interaction of NDs with cells was monitored by phase contrast live-cell imaging in real time. For both types of oxygen-terminated HPHT NDs, the cell viability and the cell number remained almost the same for concentrations up to 100 µg/mL within the whole range of ND diameters tested. The uptake of hydrogen-terminated detonation NDs caused the viability and the cell number to decrease by 80–85%. The oxidation of the NDs hindered the decrease, but on day 7, a further decrease was observed. While the O-terminated NDs showed mechanical obstruction of cells by agglomerates preventing cell adhesion, migration and division, the H-terminated detonation NDs exhibited rapid penetration into the cells from the beginning of the cultivation period, and also rapid cell congestion and a rapid reduction in viability. These findings are discussed with reference to relevant properties of NDs such as surface chemical bonds, zeta potential and nanoparticle types.

## Introduction

Carbon-based materials in the form of nanostructures are showing great promise as engineering and biomedical materials [1]. Moreover, diamond represents a new class of material with properties that are tailorable on demand [2]. This work investigates the use of diamond nanomaterials, or nanodiamonds (NDs), especially in life sciences, tissue engineering and regenerative medicine [3–6]. Diamond is biocompatible [7–8], and for advanced biomedical applications, it is particularly promising in its nanostructured forms (nanoparticles, nanostructured diamond films and composite scaffolds) [9].

ND particles can act in the single particle form (bioimaging and biosensing) [10–11], can serve as a stable delivery platform for therapeutic antibodies [12], or can be incorporated into various materials, for example, films for potential implant coatings [13]. Nanodiamond-based drug delivery has been mainly developed for advanced tumour therapies and for localized drug delivery [3,14]. Due to their stable and controllable photoluminescence, NDs are also highly promising for advanced photonic and bioimaging techniques [15–16] and for nanoscale sensing [17–18].

There are various types of NDs, but two main groups can be identified on the basis of their synthesis procedure. The first group of NDs are those synthesized by the detonation method [19], where even sub-nanometer detonation nanodiamond particles (DNDs) are produced [20]. A typical size distribution has a maximum DND diameter of around 5 nm. The second group of NDs are prepared by mechanical grinding of high-pressure high-temperature (HPHT) diamond crystals [21]. The HPHT ND particle size distribution can be mechanically controlled down to approximately 20 nm, or by further post-processing down to 1 nm, as has recently been reported [22].

NDs typically contain impurities, such as other carbon allotropes, various oxides or carbides (i.e., carriers of various functional surface groups) [3,23]. Therefore, for use in biological or biomedical studies, NDs need to be extensively purified. Numerous methods exist for removing non-diamond carbon components based on treatment with various oxidizers, such as peroxides, acids and ozone-enriched air [3,24–25]. Oxidation in air at elevated temperature is a good method for effective sp^2^ carbon removal [26–27], particle size reduction [22] and surface oxidation (i.e., the surface is covered by defined starting functional groups).

The cytotoxicity of NDs depends on their origin (i.e., DNDs or HPHT NDs), their size (distribution), their tendency to form aggregates (surface charge), the presence of impurities, and surface functionalization groups. Adverse effects on cell viability have been reported when using DNDs [24,28–32], while HPHT NDs often appear to be nontoxic [33–34]. Factors influencing the cytotoxicity of nanoparticles are their size [24,35–36] and surface functionalization [37].

In this work, we focus on cytotoxicity studies of NDs as a function of their synthesis route (DNDs versus HPHT NDs), their concentration in the medium (from 10 to 1000 mg/mL, 3 to 300 µg/cm^2^), their size (5 nm DND, 18–210 nm HPHT NDs) and their surface potential/termination (as-received and oxygen-terminated). The cytotoxicity of NDs against the SAOS-2 human osteoblastic cell line is evaluated in this work by counting adherent cells and by a mitochondrial metabolic activity test (MTS) after 3 and 7 days. Both the cell count and mitochondrial activity are positively correlated with the cell viability and are negatively correlated with the material cytotoxicity. The live-cell imaging method was used for observing the intake of NDs into the cells. The results were evaluated on the basis of particle size, surface potential, surface functional groups, and the concentration of the ND suspension.

## Results and Discussion

### Influence of particle size and concentration

**Figure 1:**
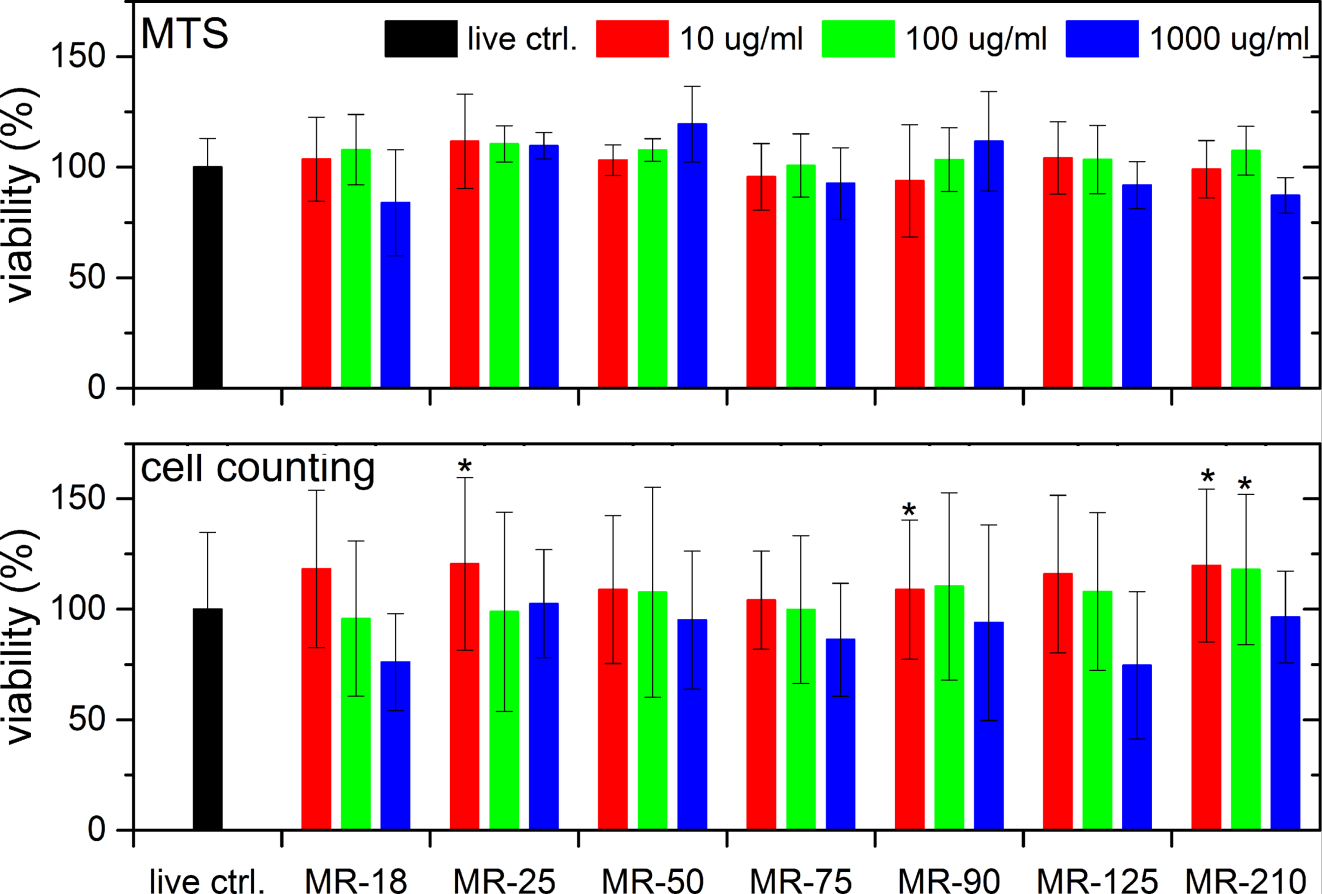
Viability of SAOS-2 cells incubated with HPHT NDs for three concentrations as a function of the mean particle diameter after 3 days. (Upper row) results of an MTS assay, (lower row) results of cell counting after cell staining. The results are given as the mean ± SD from 3 experiments, each performed in sextuplicate. ANOVA, Tukey HSD post-hoc test. “*” indicates a significant difference from MR-18 at a concentration of 1000 µg/mL (*p* < 0.05).

Figure 1 shows the results of a cell mitochondrial activity test (upper row ) and a cell nuclei count (lower row) after 3 days of cultivation for three different concentrations of 10, 100 and 1000 µg/mL (3, 30, and 300 µg/cm^2^) as a function of HPHT ND (labeled as MR-xx) particle size. Both tests proved almost no ND size or concentration dependence on cell viability after 3 days of cultivation within the measurement error. Only at the highest concentration of NDs (1000 µg/mL, 300 µg/cm^2^), the mitochondrial activity and the cell number showed a tendency to decrease in certain ND groups. However, these differences were not statistically significant. This decrease could be due to the obstruction of access to nutrients or scavenging of nutrients and growth factors from the cultivation medium by the NDs. A high ND concentration can also express chemical toxicity based on the production of reactive oxygen species.

**Figure 2:**
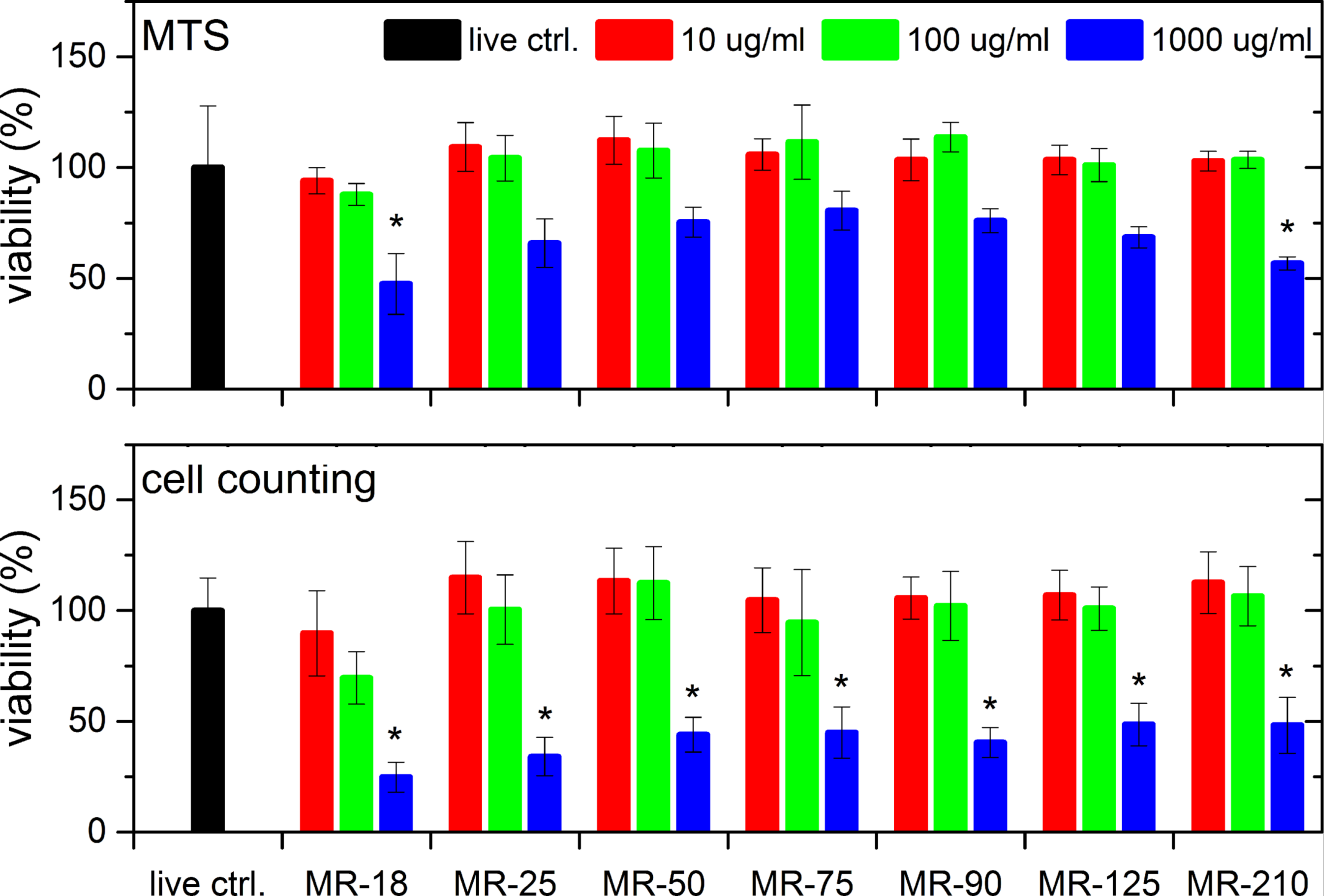
Viability of SAOS-2 cells incubated with HPHT NDs at three concentrations as a function of the mean particle diameter after 7 days. (Upper row) results of the MTS assay; (lower row) results of cell counting after cell staining. The results are given as the mean ± SD from 3 experiments, each performed in sextuplicate. ANOVA, Tukey HSD post-hoc test. “*” indicates a significant difference from MR-18 at a concentration of 1000 µg/mL (*p* < 0.05).

Figure 2 shows the results of a cell mitochondrial activity test (upper row) and counting of the cell nuclei (lower row) after 7 days of cultivation for three different concentrations of 10, 100 and 1000 µg/mL (3, 30, 300 µg/cm^2^) as a function of HPHT ND particle size. Again, no dependence of the ND size or concentration was observed for 10 and 100 µg/mL (3 and 30 µg/cm^2^) suspension concentrations after 7 days. However, a concentration-dependent toxic effect of HPHT NDs was revealed after 7 days of cultivation, where the viability of the cells cultivated in the 1000 µg/mL (300 µg/cm^2^) suspension reduced by 25% when evaluated by MTS and by 35% when evaluated by the cell counting experiment. This pronounced effect may have been caused by the previously mentioned obstruction of access to nutrients by nutrient scavenging or by reactive oxygen species. Alternatively, it could have been caused just by mechanical obstruction of the cell adhesion and division by ND agglomerates, as confirmed by live-cell imaging. A similar effect was also observed in human osteoblast-like MG 63 cells cultured in a medium with multiwalled carbon nanotubes (MWCNTs) at concentrations of 4, 40, 400, 4000 and 40000 µg/mL. On days 1, 3 and 7 after seeding, the number of MG 63 in the media with 4 and 40 µg/mL of MWCNTs was similar to or even higher than in the control cultures without MWCNTs, while at higher concentrations of MWCNTs, it decreased in a concentration-dependent manner. This was explained by the fact that MWCNTs at higher concentrations covered most of the bottom of the culture well and left only limited space for cell attachment and spreading [38].

### Influence of particle type

**Figure 3:**
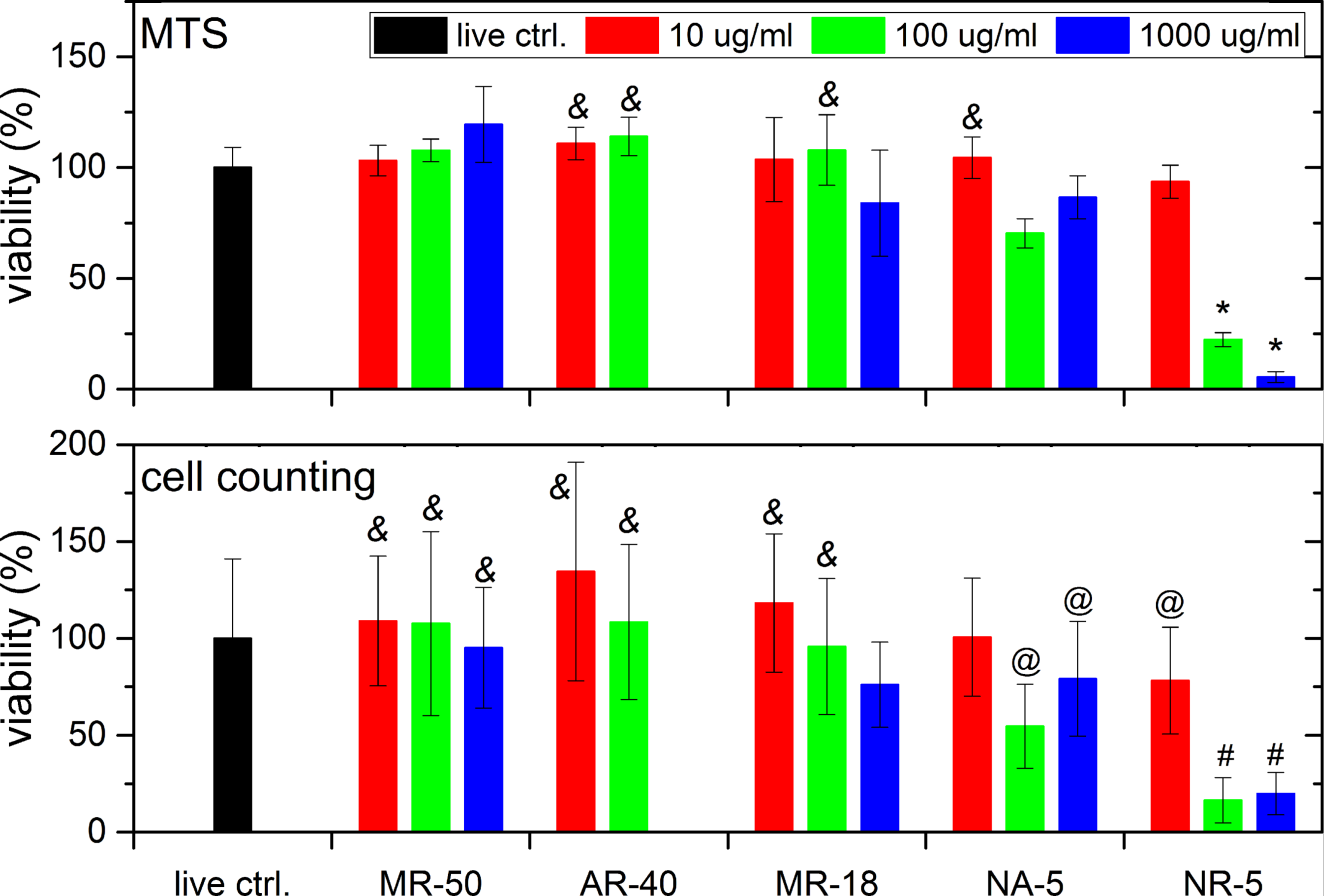
Viability of SAOS-2 cells incubated with NDs at three concentrations as a function of ND type and surface treatment after 3 days. (Upper row) results of the MTS assay; (lower row) results of cell counting after nuclei staining. The results are given as the mean ± SD from 3 experiments, each performed in sextuplicate. ANOVA, Tukey HSD post-hoc test. “&” - significant difference from NA-5 100 µg/mL (*p* < 0.05), “*” - significant difference from all other measurements (*p* < 0.01), “#” - significant difference from all other measurements except for NA-5 100 µg/mL (*p* < 0.01), “@” - significant difference from MR-50 10 and 100 µg/mL, AR-40 10 µg/mL (*p* < 0.05).

**Figure 4:**
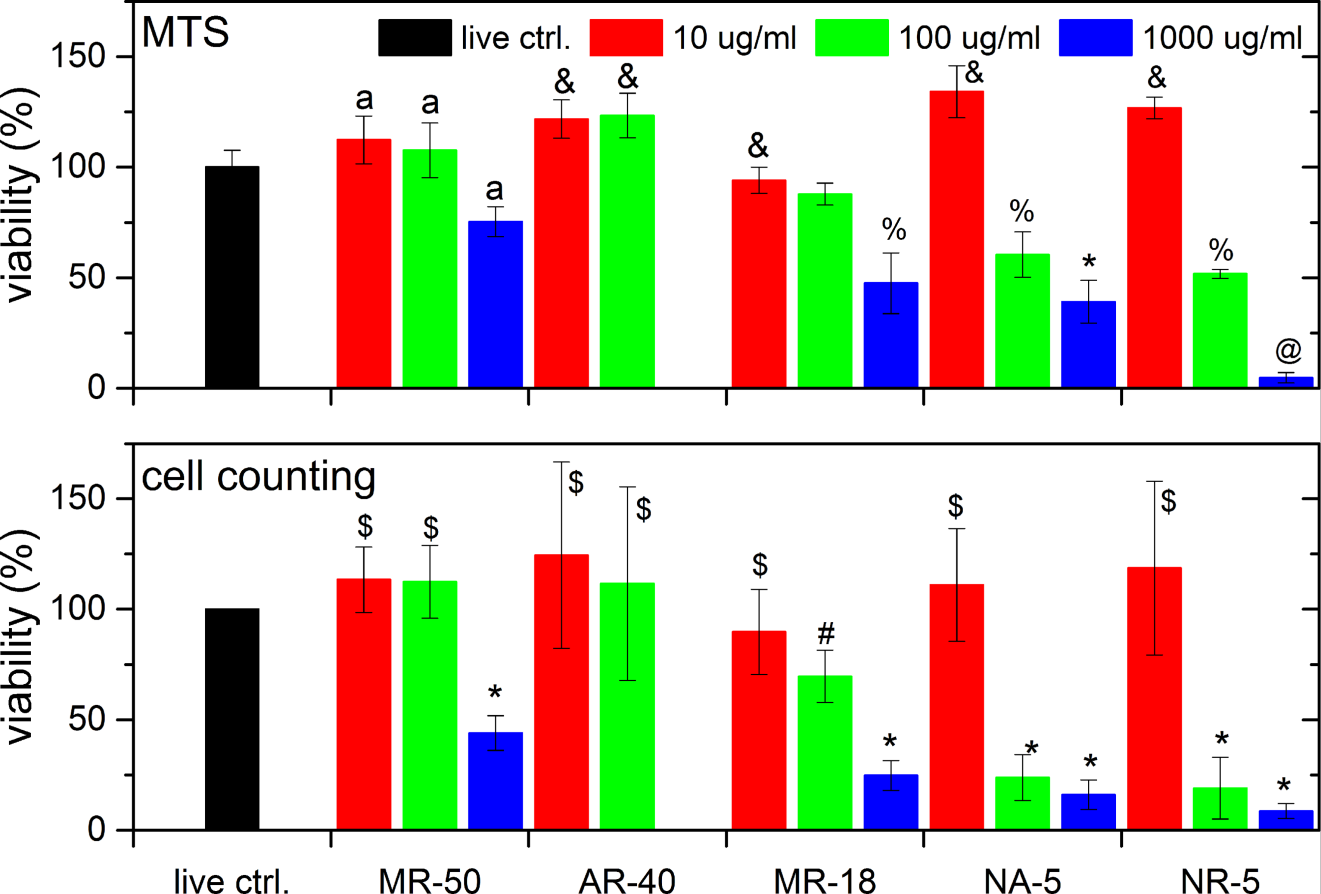
Viability of SAOS-2 cells incubated with NDs for three concentrations as a function of ND type and surface treatment after 7 days. (Upper row) results of the MTS assay; (lower row) results of cell counting after nuclei staining. The results are given as the mean ± SD from 3 experiments, each performed in sextuplicate. ANOVA, Tukey HSD post-hoc test. “a” - significant difference from NA-5 1000 µg/mL and NR-5 1000 µg/mL *p* < 0.01, “&” - significant difference from MR-18 1000 µg/mL, NA-5 100 and 1000 µg/mL, NR-5 100 and 1000 µg/mL (*p* < 0.05), “%” - significant difference from AR-40, MR-18 10 µg/mL, NA-5 10 µg/mL and NR-5 10 µg/mL (*p* < 0.05), “*” - significant difference from control, all 10 µg/mL measurements and all MR and AR 100 µg/mL measurements (*p* < 0.01), @ - same as “*” + MR-50 1000 µg/mL (*p* < 0.01), “$” - significant difference from 1000 µg/mL (*p* < 0.01), “#” - significant difference from all AR-40 and from all 100 and 1000 µg/mL concentrations, with the exception of MR-50 (*p* < 0.05).

Figure 3 and Figure 4 show a comparison between the cell metabolic activity test (upper row) and counting of the cell nuclei (lower row) for three different concentrations of 10, 100 and 1000 µg/mL (3, 30 and 300 µg/cm^2^) as a function of ND type and surface treatment after 3 days (Figure 3) and 7 days (Figure 4) of cultivation. Three ND types with differing characteristics were selected: non-luminescent HPHT NDs of two diameters as described in the previous section (MR-18 and MR-50), HPHT NDs with photoluminescent nitrogen-vacancy (N-V) centers (AR-40), and detonation NDs with hydrogen termination (NR-5, as-received) and with oxygen termination (NA-5, annealed).

First, we compared the photoluminescent HPHT NDs (AR-40) with non-luminescent HPHT NDs of similar size (MR-50) and of smaller size (MR-18). The photoluminescent NDs AR-40 were available only in two concentrations: 10 and 100 µg/mL (3 and 30 µg/cm^2^). Both HPHT ND types were oxidized. Neither of the HPHT NDs are cytotoxic in low and medium concentrations after 3 and 7 days of cultivation. A visible decrease in cell viability can be observed with the highest ND concentration of MR-50 and 18. The decrease was statistically insignificant after 3 days (Figure 3). However, after 7 days the cell viability had decreased significantly (Figure 4) in comparison with the control cells in the pure culture medium and in comparison with the other concentrations of MR. The similar cytotoxicity of these HPHT NDs is probably caused by the similar production method, which results in oxidized surface termination and surface energy of the NDs. The presence of photoluminescent N-V centers had no negative effect on cell viability in the concentrations studied (3 µg/cm^2^ and 30 µg/cm^2^).

Similar to the other HPHT NDs with an oxidized surface, the NDs with N-V optical centers expressed no significant toxicity when compared with the ND-free living control (i.e., cells grown in polystyrene wells in a medium without diamond nanoparticles). Similar results were also obtained in a study by Vaijayanthimala et al. [11], in which the proliferation of HeLa cells and 3T3-L1 pre-adipocytes exhibited no significant difference in cultures exposed and unexposed to photoluminescent nanodiamonds. This positive effect can be attributed to the fact that the mechanism of the ND uptake was clathrin-mediated endocytosis, that is, a physiological cellular mechanism for internalization of various bioactive substances from the extracellular environment. The negligible difference in cytotoxicity is caused by the similar production method, surface termination and energy of the NDs.

The surface termination of the two HPHT ND types is similar, despite the fact that the photoluminescent NDs were further postprocessed utilising methods influencing the bulk of the NDs (formation of vacancies, and N-V sites supported by high-temperature annealing in vacuum) [39]. This was confirmed by the fact that there was no obvious difference in the FTIR spectra between the MR-18 sample and the AR-40 sample. The zeta potential of MR-type NDs were negative, typically in the range of −20 to −40 mV [40–41], comparable with the −37 mV zeta potential value of the AR-40 sample. The stock concentration of AR-40 did not allow us to test the effect of the highest ND concentration (1000 µg/mL, 300 µg/cm^2^).

Next, we compared the influence of surface treatment by evaluating NR-5 and NA-5 samples (i.e., samples of detonation NDs as-received and treated by annealing, respectively). These detonation NDs in their as-received state have a positive zeta potential, which is characteristic for hydrogenated NDs. The XPS analysis indicated a mixture of hydrogen and oxygen states on the surface. They were fully oxygenated by annealing at high temperature. The viability of the cells cultivated with NR-5 (100 and 1000 µg/mL, 30 and 300 µg/cm^2^) had already reduced by 80–85% after 3 days of cultivation. However, the annealing of these DNDs (NA-5) reduced their toxicity significantly by 30%. This effect could still be observed after 7 days of cultivation, where the cytotoxicity of air-annealed DNDs decreased by 25% for a 100 µg/mL (30 µg/cm^2^) suspension, and by 30% for a 1000 µg/mL (300 µg/cm^2^) suspension. The particle size should be same as these samples are produced from the same batch of DND powder. The main difference lies in the surface termination and energy. The surface termination differs mainly in antisymmetric and symmetric CH_2_, CH_3_ (decrease), C═O (increase) and strong overlapping C–O, C–C bonds with air oxidation [42]. The oxidation of DNDs has a strong impact on their zeta potential, which influences the aggregation or the selection of adhered proteins from the culture medium. The as-received DNDs have a strongly positive zeta potential of ≈40 mV, while oxidation reverses it to approximately −40 mV [41].

The cell surface charge is influenced by the actual biochemical composition of the cytoplasmic membrane and the state of the cell. It is an important biophysical parameter influencing the interaction with the cell surroundings.

The cell surface charge (zeta potential) of human cells was between −20 and −30 mV caused by the presence of nonionogenic groups within phospholipids, proteins, and their polysaccharide conjugates [43]. Thus, we can expect similar zeta potential values for SAOS-2 cells, which are comparable with HPHT NDs and annealed DNDs, (i.e., negatively charged nanoparticles). It is known that negatively charged nanoparticles are less attractive for binding to the cell membrane than positively charged nanoparticles, which can be internalized more rapidly [44]. Positively charged nanoparticles have been reported to improve the efficacy of imaging, gene transfer and drug delivery. However, at the same time, negative effects like impaired integrity of cytoplasmic membrane and damage of other membranous organelles like mitochondria and lysosomes were observed. Also, more autophagosomes were produced by the cells cultivated with positively charged nanoparticles ([45] or for a review see [46]). Hydrogenated positively charged ND particles impaired the radio-resistance of cancer cells and potentiated radiation-caused DNA damage and the generation of cytotoxic reactive oxygen species [47]. Thus, the positive charge of our as-received DNDs could, at least partly, explain their more pronounced cytotoxic effect than that observed in negatively charged annealed DNDs.

Finally, we can compare the NDs produced by mechanical grinding and by the detonation method, MR-18 and NR-5, respectively. Here, the main differences are in the production method and the particle size (18 nm and 5 nm), while the effect of surface termination is minimized due to air annealing [42]. We have shown that air annealing of as-received DNDs reduced mainly bands in the 2800–3000 cm^−1^ region corresponding to CH_2_ and CH_3_ stretching vibrations, and they give rise to a C═O stretch at 1775 cm^−1^, C–O stretch at 1294 cm^−1^, and a C–O–C stretch at 1077 cm^−1^.

This produces surface termination similar to that of the as-received HPHT NDs (compare Figs. 1 in [42] and [40]). The zeta potentials have almost the same value of −40 mV for both ND types [41]. This explains the similarity in cytotoxic behaviour, where annealing of DNDs reduced their toxicity significantly. A decrease in the cytotoxicity is still observable after 7 days of cultivation (a 40% reduction), likely because some CH_2_, CH_3_ and C–H stretch bands remain and some differences in particle diameter. Finally, the dependence of the concentration on the cell viability was again most pronounced for the highest concentration (1000 µg/mL, 300 µg/cm^2^).

### Live-cell imaging of diamond nanoparticle uptake

Live-cell imaging (see Supporting Information File 1 and Supporting Information File 2 for full experimental data) confirmed the formation of DND aggregates in the suspension (10 µg/mL, 3 µg/cm^2^). Particle aggregates are collected by cells from their surroundings. DNDs then accumulate on their surface and are endocytosed during the experiment. The accumulation and the cytotoxicity of the DNDs in the cell depends on their surface termination.

Live-cell imaging of SAOS-2 cells with as-received DNDs (NR-5, see Supporting Information File 1) reveals rapid uptake of NDs by the cells. The DNDs penetrated the cells early in the experiment. The video begins with six living cells. The cells are soon congested by the nanoparticles then their viability drops rapidly. Five cells undergo cytokinesis, but at least half of the cells are dead at the end of the experiment. In at least one or two of the cases of cell death, an expelled cytoplast can be seen exiting the cell membrane. This indicates uncontrolled cell death and rupture. The remaining living cells have an elongated shape due to the accumulated NDs which mechanically restrain their spreading and migration [38].

Unlike NR-5, the air-annealed DND aggregates (NA-5, see Supporting Information File 2) are not taken up by the cells as rapidly as their non-annealed counterparts. The video starts with a similar number of living cells as described above. The cells undergo 14 cytokineses, and most of them survive until the end of the video sequence. Only two or three cell death events can be recognized in the video.

### Photoluminescence of diamond nanoparticles

**Figure 5:**
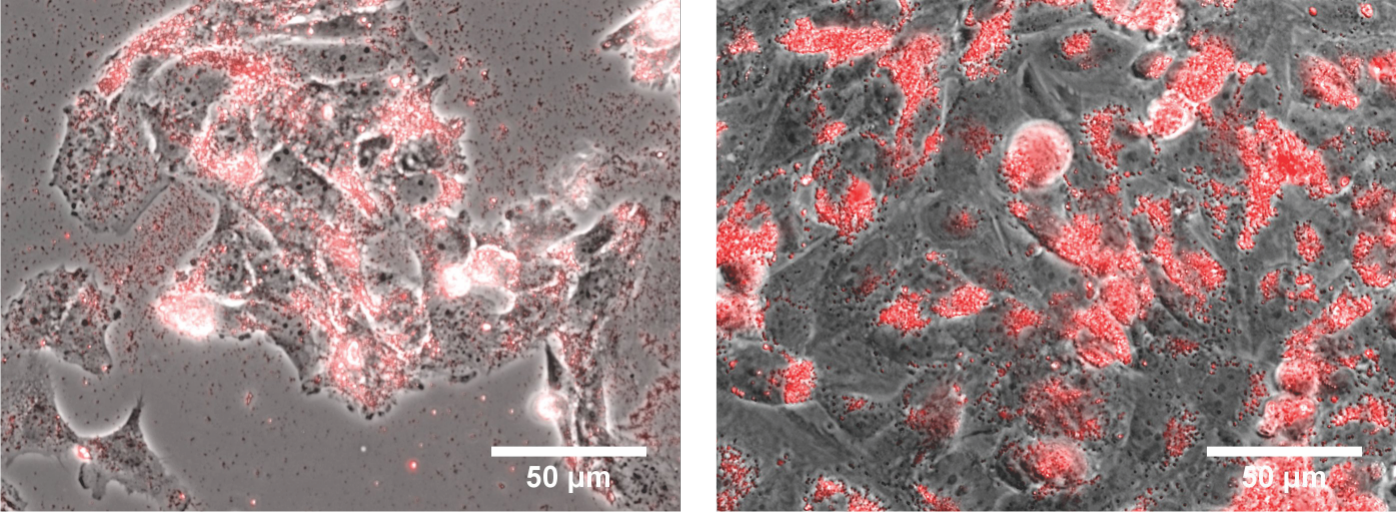
Phase contrast images of photoluminescent NDs (AR-40, 100 µg/mL) incubated with SAOS-2 cells after 3 days (left) and after 7 days (right).

Figure 5 shows ﬂuorescence phase contrast images of osteoblasts and photoluminescent NDs (AR-40, 100 µg/mL, 30 µg/cm^2^) grown for 3 and 7 days. The images reveal the presence of NDs in the cells. The red photoluminescence signal around the outside of the cell nuclei indicate that the NDs could not penetrate the nuclear envelope and stay in the cytoplasm. A certain amount of NDs are present outside the cells, probably in the loose serum protein/ND aggregates or in cell debris. The well-spread osteoblasts are homogeneously and conﬂuently distributed. Good cell adhesion and division confirms the viability of the cells with photoluminescent NDs (AR-40).

## Conclusion

A comparison of cell viability with various types of NDs (HPHT, HPHT PL, DND) and surface termination and energy showed that the toxicity is mostly dependent on particle surface modification. In our study, oxygen termination emerges as the best surface modification for ND particles from the point-of-view of their biocompatibility. The viability of cells cultivated with positively charged as-received DNDs (100 and 1000 µg/mL, 30 and 300 µg/cm^2^) had already decreased after 3 days of cultivation. However, annealing these NDs reduced their toxicity significantly. This effect could still be observed after 7 days of cultivation.

A concentration-dependent toxic effect of HPHT NDs was revealed after 7 days of cultivation when the viability of the cells cultivated at a 1000 µg/mL (300 µg/cm^2^) concentration reduced by 25–30% in comparison with 100 µg/mL and 10 µg/mL (30 µg/cm^2^ and 3 µg/cm^2^). This was associated with the mechanical obstruction by NDs preventing cell adhesion, migration and division.

In comparison with the results of our previous studies, the air annealing of as-received DNDs reduced bands mainly corresponding to CH_2_ and CH_3_ stretching vibrations, and gave rise to C═O, CO and C–O–C stretch bands [41–42]. The zeta potential was also reversed from positive values to negative values. Thus the surface state of as-received DNDs is rendered similar to the state of HPHT NDs with similar cytotoxicity results. A decrease in viability was still observable after 7 days of cultivation, mostly due to the remaining CH_2_, CH_3_ and C–H stretching bands. The dependence of concentration on cell viability was again observed.

The comparison of the viability of the cells cultivated with various types of NDs indicates that the toxicity of NDs is probably dependent on the surface functional groups and the zeta potential rather than on the diameter of the particles.

Live-cell imaging showed that as-received DNDs penetrated rapidly into cells and caused rupture. O-terminated DNDs aggregated into large clusters, and the cells internalized them at a slower rate with a low impact on their viability for the first 3 days. After 7 days of cultivation, the MTS test revealed lower cell viability for ND concentrations of 100 µg/mL and 1000 µg/mL (30 µg/cm^2^and 300 µg/cm^2^). Nevertheless, O-termination increases the biocompatability of diamond nanoparticles and can be considered an advantageous modification.

## Experimental

### Origin and surface modification of diamond nanoparticles

Nanodiamond particles produced by two different methods were used: NanoAmando DNDs (NanoCarbon Research Institute, Japan) with a nominal diameter of 5 nm, HPHT NDs (Microdiamant AG, Switzerland) with median sizes from 18–210 nm, and 40 nm HPHT NDs with N-V-based photoluminescence (Adámas Nanotechnologies, USA). The particles were used either as-received or were oxidised by air annealing at 450 °C for 30 min [48]. For the detonation nanodiamonds purchased from NanoCarbon, the as-received nanodiamonds had a mixture of hydrogen and oxygen states on the surface with a positive zeta potential (characteristic for hydrogenated NDs). A fully oxidized state was achieved by air annealing, which resulted in oxygen-termination. The other diamond nanoparticles, namely high-pressure high-temperature (HPHT) DNPs and photoluminescent HPHT NDs, were oxidized in their as-delivered state.

### Characterization of diamond nanoparticles

The size of the ND particles and their zeta potential was determined by dynamic light scattering (DLS) measurements in water at 25 °C using a Nano-ZetaSizer (Malvern, UK) equipped with an He-Ne laser. A separate disposable folded capillary cell was used for each set of ND measurements to eliminate sample cross contamination.

A Nicolet 8700 FTIR spectrometer (Thermo Scientific, USA) was equipped with N_2_ purging, a KBr beamsplitter and an MCT detector cooled by liquid nitrogen. 50 µL of the water suspension with NDs was applied on the Au mirror by the drop-casting method just prior to the grazing angle reflectance FTIR measurements. The optical absorbance was calculated in standard absorbance units as *A =* −log(*R*/*R*_0_), where *R* is the spectrum measured with NDs and *R*_0_ is the reference (background) spectrum recorded using the clean Au mirror before the NDs were applied. In all cases, the spectra represent an average of 128 scans recorded with a resolution of 4 cm^−1^.

The basic characteristics and the notation of the NDs that were used are summarized in Table 1. More data on ND characterization by FTIR and XPS can be found in our previous works [42,49].

**Table 1:** Characteristics and notation of diamond nanoparticle (ND) samples.

ND type	Particle size [nm]	Treatment	Notation

HPHT NDs	18	as-received	MR-18
	25		MR-25
	50		MR-50
	75		MR-75
	90		MR-90
	125		MR-125
	210		MR-210
DNDs	5	as-received	NR-5
	5	annealed	NA-5
HPHT PL NDs	40	as-received	AR-40

### Evaluation of cell viability

The cell viability upon exposure to the NDs was evaluated using the SAOS-2 human osteoblastic cell line (European Collection of Cell Cultures, Salisbury, UK, Cat. No. 89050205). The SAOS-2 cell line was used for biocompatibility experiments based on cell anchorage dependency and homogeneity. Two complementary methods were used. The first method was based on the mitochondrial metabolic activity test (MTS), while the second method was based on counting adherent cells. The cells were cultivated in the recommended McCoy’s 5A medium (Sigma-Aldrich, USA) with 15% fetal bovine serum (FBS, Sigma-Aldrich, USA). The cells were seeded at a density of 15000 cells/cm^2^ in a sterile 96-well plate (TPP, Switzerland) and were cultivated for 24 h before the nanoparticles were added. Cells from the 82nd passage to the 86th passage were used for the experiments. The NDs were sonicated in a UP 100H sonicator (Hielscher, Germany) in sterile distilled water in a stock concentration of 10 mg/mL, at 80 W for 30 min. The stock ND suspension was sterilized by exposure to UVC light (Esco germicidal lamp, spectrum peak at 253.7 nm) for 30 min. The working concentrations of 10, 100 and 1000 µg/mL were then diluted in the cultivation medium with FBS. The original medium was aspired off the cells, and the medium with NDs was pipetted onto the cells. The cells were cultivated with the NDs for 3 and 7 days under standard cultivation conditions (37 °C, 5% CO_2_). The cell metabolic activity (a marker of cell viability and growth) was measured using the colorimetric MTS test (CellTiter 96**^®^** - Promega, USA). The absorbance of the colorimetric MTS test was measured at 490 nm, and a reference measurement was taken at 650 nm. This experiment was repeated three times in sextuplicate for each experimental group. The cells on the samples were then washed with phosphate buffered saline and were fixed with 4% paraformaldehyde for 10 min. The nuclei of the fixed cells were then stained using Hoechst 33258 dye for cell counting. Micrographs of the stained nuclei were acquired using an IX71 microscope (Olympus, Japan) with a 10× lens. 12 micrographs were taken for each sample. The cell nuclei were automatically counted using open source ImageJ image processing software. Photoluminescence microphotographs of fixed cells co-cultivated with photoluminescent NDs (AR-40), 100 µg/mL (30 µg/cm^2^), were also taken using an IX71 microscope (Olympus, Japan) with a 40× lens. The AR-40 NDs were excited by green excitation light, and their red photoluminescence was collected through a U-MWG2 filter cube (Olympus, Japan).

The statistical significance of the differences in the cell metabolic activity and in cell number among the samples was evaluated using ANOVA with the Tukey HSD post-hoc test.

### Live-cell imaging

The live-cell imaging method was used for observing the ND uptake in the cells. Live-cell imaging was again performed on the SAOS-2 cell line. The cells were seeded on a 35 mm diameter Petri dish with a 0.17 mm glass bottom and were cultivated for 24 h. The ND suspension was prepared in the same way as for the viability test. For live-cell imaging, only 10 µg/mL and 100 µg/mL concentrations were used. The live-cell imaging was performed on the TE2000 microscope with 40× magnification, (Nikon, Japan), Plan Fluor, ELWD objective (Nikon, Japan) with phase contrast installed. The microscope was equipped with a cell incubation chamber (Solent Scientific, UK) with humidity regulation (95%), controlled temperature (37 °C) and CO_2_ concentration (5%). The cells were photographed for 72 h at 2 min intervals for the first 6 h and thereafter at 10 min intervals.

## Supporting Information

File 1 Live-cell imaging of NR-5 (10 µg/mL, 3 µg/cm^2^) uptake by the SAOS-2 cells.

File 2 Live-cell imaging of NA-5 (10 µg/mL, 3 µg/cm^2^) uptake by the SAOS-2 cells.

File 3 FTIR comparison of MR-18 and AR-40 nanodiamonds.

File 4 Live-cell imaging of SAOS-2 cells after three days without nanodiamonds.
