# Supplementary figures and images for: Uptake and intracellular accumulation of diamond nanoparticles – a metabolic and cytotoxic study
Source: Beilstein J Nanotechnol. 2017 Aug 10;8:1649–57. doi: 10.3762/bjnano.8.165 (PMC5564261; doi:10.3762/bjnano.8.165)

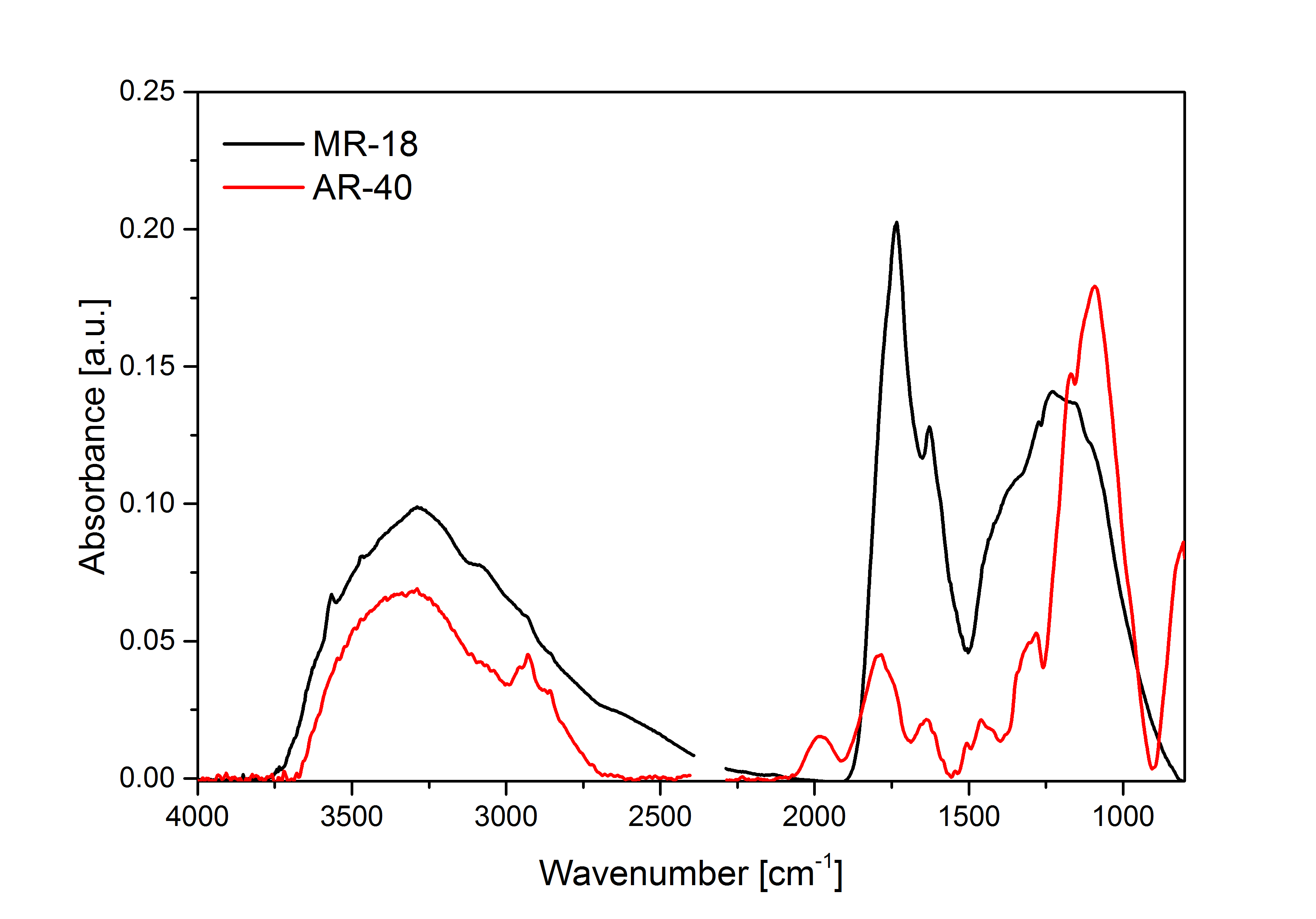

Supplement: File 3 — FTIR comparison of MR-18 and AR-40 nanodiamonds. [file Beilstein_J_Nanotechnol-08-1649-s003.png]
